# Positive effects of fast growth on locomotor performance in pelagic fish juveniles

**DOI:** 10.1007/s00442-022-05216-6

**Published:** 2022-07-04

**Authors:** Masahiro Nakamura, Michio Yoneda, Taizo Morioka, Akinori Takasuka, Nozomi Nishiumi

**Affiliations:** 1 Fisheries Technology Institute, Japan Fisheries Research and Education Agency, Hakatajima Filed Station, Imabari, Ehime 794-2305 Japan; 2 Graduate School of Agricultural and Life Sciences, The University of Tokyo, Bunkyo, Tokyo 113-8657 Japan; 3 National Institute for Basic Biology, Higashiyama 5-1, Myodaiji, Okazaki, Aichi 444-8787 Japan

**Keywords:** Growth rate, Locomotor performance, Burst speed, Trade-off, Pelagic fish

## Abstract

Many laboratory experiments on aquatic vertebrates that inhabit closed water or coastal areas have highlighted negative effects of fast growth on swimming performance. Nonetheless, field studies on pelagic fishes have provided evidence of survival advantages of faster-growing individuals. To reconcile this contradiction, we examined the relationship between growth rate and swimming performance as a continuous function for juveniles of chub mackerel (*Scomber japonicus*) using 3D tracking analysis. For experiments, 20, 24, 27, and 30 days post-hatch individuals within the size range of 14.5–25.3 mm were used. We found that the growth–swimming (burst speed) relationship in chub mackerel was substantially positive and it was supported by morphological traits such as muscle area, which were also positively related with growth rate. This finding is consistent with field observations showing selective survival of fast-growing individuals of this species, reconciling the current contradiction between laboratory experiments and field observations. A dome-shaped quadratic curve described the relationship between growth rate and burst speed better than a linear or cubic function, suggesting that growth may trade-off with swimming performance, as reported in many previous studies, when it is extremely fast. These results, obtained from the rarely tested offshore species, strongly suggests the importance of experimental verification using animals that inhabit various types of habitats in understanding the principles underlying the evolution of growth–locomotor relationship.

**Supplementary Information:**

The online version contains supplementary material available at 10.1007/s00442-022-05216-6.

## Introduction

Somatic growth is a fundamental biological factor that affects directly the rate of increase of an individual’s body size from an ovum to a multicellular reproductive adult, and indirectly the survival. Not surprisingly, fast growth is often perceived as beneficial, especially in the early life stages, because it contributes to rapid development of vital organs and early acquisition of the potential benefits of large size (Arnott et al. 2006). Despite this assumption, growth is commonly observed to occur at rates lower than the maximum that is physiologically possible (Dmitriew 2011). This is because growth itself imposes a significant physiological cost, which results in trade-offs between growth and other essential life history traits (reviewed elsewhere, e.g. Arendt 1997; Scharf et al. 2009; Dmitriew 2011). Swimming performance of aquatic vertebrates is one of the well-known traits that trade-off with growth rate, and most studies reported negative effects of accelerated growth on swimming performance. These negative relationships were observed through experimental studies on laboratory animal species, mainly Atlantic silverside (*Menidia menidia*) (Billerbeck et al. 2001; Munch and Conover 2004), three-spined stickleback (*Gasterosteus aculeatus*) (Lee et al. 2010, 2016) and tadpoles (Arendt 2003).

However, these negative effects of growth on swimming performance contradict with the positive effects of growth observed in many field studies on fishes, especially pelagic fishes that inhabit offshore areas. Numerous field studies reported evidence of selective survival of fast-growing individuals during their larval and/or juvenile stages (Hare and Cowen 1997; Allain et al. 2003; Oozeki et al. 2003; Takasuka et al. 2003, 2004, 2007; Kamimura et al. 2015; Taga et al. 2019; Khamassi et al. 2020). This sheds light on the possibility that growth rate may positively correlate with swimming performance in these species. This is because predation is considered to be one of the determining factors in larval survivorship of pelagic fishes (Bailey and Houde 1989; Hallfredsson and Pedersen 2009), and swimming performance, especially burst speed, is likely to play a key role in predation avoidance for small fishes (Walker et al. 2005).

Relationships between growth rate and swimming performance may vary among habitat types. Most studies on growth–swimming relationships used aquatic vertebrates that inhabit closed water or coastal areas including estuaries, which greatly differ from offshore waters in environmental characteristics. The greatest difference may be the presence/absence of a shelter or turbid zone in which they take refuge to avoid predation. Such places are often found in coastal areas or lakes and ponds, but not in offshore areas, suggesting that physical performance is a key factor in determining success or failure of predation avoidance in offshore areas. The importance of a particular trait in survivorship will differ among habitat types, and habitat-specific selection pressures may lead to the evolution of different relationships between growth rate and a specific trait. In fact, a previous study on three-spined stickleback reported that the existence of trade-offs between growth rate and swimming performance depended on their habitats (Álvarez and Metcalfe 2007). However, no study has yet reported a positive relationship between growth rate and swimming performance in a way that excludes the effect of body size or any other possible contributing factors. Thus, the contradiction in the relationship between growth and survivorship remains unsolved.

In the present study, we tested a hypothesis that a positive correlation between growth rate and swimming performance has evolved in pelagic fish, which spend most of their life in the open ocean. We used chub mackerel (*Scomber japonicus*) juveniles as a model species to test the growth–swimming relationship, since faster-growing individuals were found to survive better in the outer sea (Kamimura et al. 2015; Taga et al. 2019). Individuals within a limited size range with various values of growth rate were used for analysis to reduce the effect of body size. Routine speed and burst speed, which reflect foraging behavior and escaping behavior (Fuiman and Cowan 2003), respectively, were measured as indicators of swimming performance. Morphological traits which can affect locomotor performance were also measured.

## Materials and methods

### Egg hatching and larval rearing

Eggs were obtained from induced spawning of captive broodstock maintained at the Hakatajima Station, National Research Institute of Fisheries and Environment of Inland Sea (Imabari, Japan), following the procedure of Nyuji et al. (2012). To obtain an adequate number of eggs, we used two parent groups of different ages, 1 year old and 3 years old. Ten individuals of 1-year-old fish and three individuals of 3-year-old fish of each sex were injected intramuscularly with 400 μg kg^–1^ of body weight gonadotropin-releasing hormone analogue (GnRHa) on 17-May-2019 and were maintained in 50,000-L (3-year-old individuals) and 20,000-L (1-year-old individuals) square tanks with circulating seawater. Eggs spawned on 16-June and 18-June were sampled for experiments. Approximately 10,000 eggs collected evenly from the two parental age groups were pooled in a 1000-L plastic tank which was maintained at an average temperature of 22 °C, and a total of four such tanks were prepared. The eggs were incubated under a photoperiod cycle of 14 h light and 10 h dark. The larvae were fed with a mixture of rotifers (*Brachionus*) and planktonic algae (*Isochrysis galbana* or *Nannochloropsis oculata*) once per day until 10 days post-hatch (dph), after which they were also fed with newly hatched brine shrimp (*Artemia salina*) once per day and reared until 30 dph. Ration level was kept high (rotifers: 20 individuals/mL; brine shrimp: 0.5 individuals/mL) throughout the experimental period to make sure that unconsumed food always remained in the tanks, so as to keep the feeding regime ad libitum for all individuals in the population and prevent feeding competition.

### Sampling of juveniles of different growth rates

**Fig. 1:**
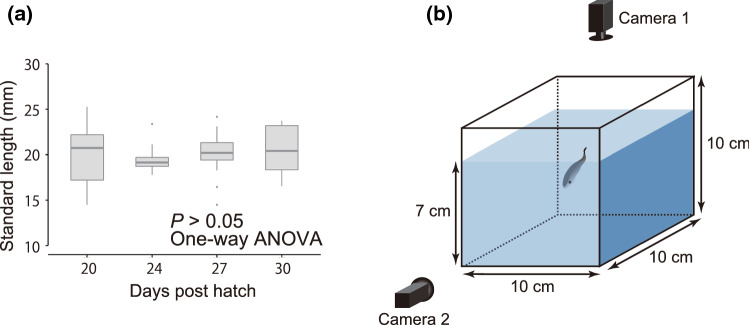
Size distribution of 57 individuals tested at different days post-hatch (**a**) and schematic diagram of the experimental tank (**b**)

Juveniles were sampled from the rearing tanks at 20, 24, 27, and 30 dph for use in behavioral assays. Specifically, we collected some of the largest individuals from the rearing tanks in the first sampling. In the second to fourth samplings, we selectively extracted individuals within substantially the same size range as the first sample. Since the feeding regime was ad libitum for all individuals in the rearing tanks, this sampling protocol ensured that the earlier samples represented the faster-growing fish and the later samples represented the slower growing fish in the experimental population, reflecting inherent differences in the growth physiology of population members. The standard length (SL) of the experimental fish ranged from 14.5 to 25.3 mm (Fig. 1a, see Table S1 for details).

### Behavioral assays

The sampled juveniles were used for two behavioral assays: (1) a routine swimming assay in which the spontaneous swimming behavior of individual fish was monitored in the absence of any stimulus, and (2) a startle stimulus assay in which a glass probe was slowly approached toward a fish’s head and the triggered startle response was monitored. Approaching an object toward fish such as a probe or a simulated predator is a common technique for eliciting a startle response from the fish (Batty and Blaxter 1992; Billerbeck et al. 2001; Fisher et al. 2007). Data from the routine swimming assay and the startle stimulus assay were used for determining the routine speed and the burst speed, respectively (see below).

For an experimental tank for these assays, a transparent acrylic cube (10 cm × 10 cm × 10 cm) was used, filled with seawater to 7 cm height. Two cameras (Grasshopper3, FLIR Integrated Imaging Solutions Japan Ltd., Japan) were installed above the tank and adjacent to a side of the tank (Fig. 1b). The bottom and the three sides not facing the camera 2 were covered with white acrylic plates to improve the visibility of fish (not illustrated in Fig. 1a). In each trial of the behavioral assays, a single fish was introduced in the tank and left for 10 min for acclimation to the tank. Then, the spontaneous swimming behavior of the fish was recorded for 20 s by the two cameras, which was repeated three times at 1-min intervals (routine swimming assay). Next, another 20-s recording was conducted, during which the probe was approached to the fish to draw a startle response. The recording was repeated three times at 5-min intervals, which allowed the fish to recover (startle stimulus assay). All trials were recorded at 20 fps. All fish were humanely killed with an overdose of 0.1% 2-phenoxyethanol, and their SLs were measured. Body depth (hereafter referred to as BD) and muscle area (hereafter referred to as MA) were measured using a frame of video data from the camera 2 in which the fish body was orthogonally oriented to the camera 2. Following Fisher and Hogan (2007), we defined BD and MA as “height at the deepest region of the fish body excluding the head” and “area excluding the fins and the head and gut region”, respectively.

### Data processing

For deriving the 3D coordinates of fish, we first synchronized video frames from the two cameras. This synchronization was primarily carried out by pairing those two cameras with a GPIO cable. Then we manually checked and corrected synchronization errors of 1–3 frames that incidentally occurred. From these synchronized images, the 3D location of fish was estimated on the basis of optical principles, using rays between fish and the two cameras. Refraction of rays at the water surfaces was taken into account, whereas refraction at the transparent acrylic panels of the experimental tank was ignored because they were thin enough not to cause significant optical effects. We first calculated the part of the ray that was in the air, between each camera and the water surface. This ray was calculated from the location of each camera, the 2D pixel coordinates of fish in the video, and reference points with known 3D locations and 2D pixel coordinates. We then geometrically calculated the remaining part of each ray, which was in the water, by tracing a line from the intersection on the water surface with a refracted angle under Snell’s law, which should be in the direction of the fish. Through these processes, we obtained two rays in the water from the two cameras, and the estimated 3D location of the fish was computed as the point where these two rays crossed. The mean and maximum shifting errors of the estimation method were 0.76 mm and 1.73 mm, respectively, against 80 test objects located inside the experimental tank. The 2D pixel coordinates of fish were automatically obtained with UMATracker (Yamanaka and Takeuchi 2018).

Routine speed and burst speed were calculated from time-series data of 3D coordinates of fish in the routine swimming assay and the startle stimulus assay, respectively. The routine speed of an individual in each 20-s recording period was defined as the mean speed for the entire period. The burst speed of an individual in each 20-s recording period was defined as the maximum speed in any of the four frames (i.e. 1/5 s) after the initiation of a burst event, which was defined as a startle response initiated in a C- or S-type start (Domenici 2010) following the approach of the probe. Growth rate was calculated for each individual by subtracting the SL of hatched larvae (fixed at 3 mm) from the individual’s SL and dividing the resultant value by dph.

### Statistical analysis

All the statistical procedures were conducted using the statistical software R version 4.1.2 (R Core Team 2021). No significant difference was found in SL among the observed fish of different dph (One-way analysis of variance; *P* > 0.05) (Fig. 1a). Therefore, data from all individuals were used for the following analysis. To clarify whether SL and growth rate had a significant effect on routine and burst speeds, generalized linear mixed models (GLMMs) were applied. Effects of SL and growth rate were tested in the same model because no severe multicollinearity was detected (Tables S2, S3). Otolith cores of some fish were marked with alizarin complexone (ALC) as part of a different study. Therefore, the effect of ALC marking was also tested. The models contained the following variables and random effects: the response variable was routine speed or burst speed; explanatory variables were SL, growth rate, and ALC marking (categorical variables: marked and non-marked); random effects were individual ID and rearing tank. Since growth rate appeared to have a major effect on burst speed, we tested up to third-order linear models to describe the relationship between burst speed and growth rate. Three GLMMs using linear, quadratic, and cubic polynomials, which contained growth rate as an explanatory variable and individual ID and rearing tank as random effects, were fitted to the same data as the former GLMM analysis.

In addition, two generalized linear models (GLMs) and a GLMM were applied to determine whether there were any significant relationships between morphological traits and growth rate or burst speed. At first, the significance of the relationships between morphological traits and growth rate was examined by GLM analysis. In this analysis, the response variable was growth rate and explanatory variables were MA and BD. Since a significant relationship was detected between growth rate and MA, the relationship between MA and dph was determined by a GLM function, which contained MA as a response variable and SL and dph as explanatory variables. Finally, the relationship between MA and burst speed was examined using a GLMM function, which contained burst speed as a response variable, MA as an explanatory variable, and individual ID and rearing tank as random effects. No severe multicollinearity was detected in any of the GLM analyses (VIF < 5.48). The “performance version 0.4.6”, “multcomp version 1.4.13”, and “glmmTMB version 1.1.2.3” packages were used for checking multicollinearity, determining the difference in SL between age groups, and GLMM analysis, respectively. All of the GLMM and GLM analyses were based on a Gamma distribution and the significance level of all the statistical tests was set at *α* = 0.05. Sample sizes were 171 and 161 for the GLMM analyses of routine speed and burst speed, respectively, and 57 for the GLM analyses.

## Results

### Relationships between growth rate and swimming performance

**Fig. 2:**
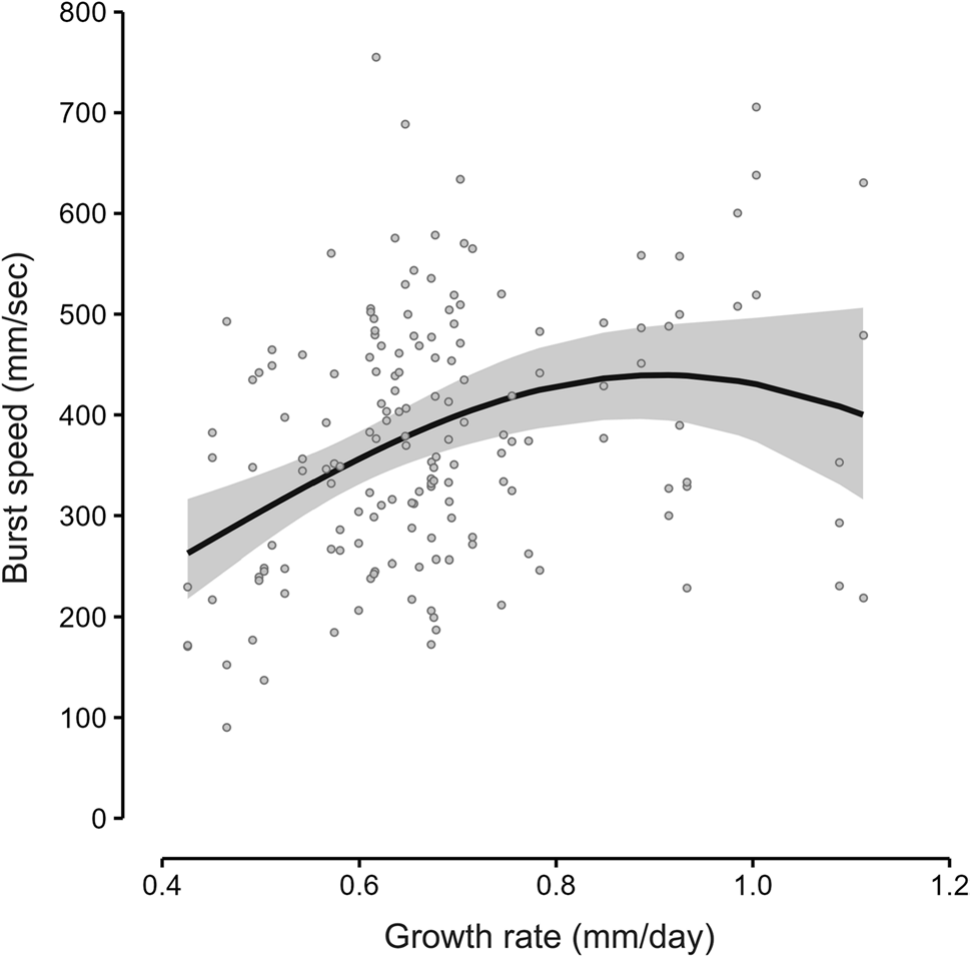
Relationship between growth rate and burst speed. Solid line indicates the quadratic curve fitted to the data (*n* = 161). Gray shadings on either side of the lines indicate the 95% confidence interval

The GLMM analyses showed that routine speed was not significantly affected by either SL or growth rate (Table 1; see Table S2 for details). In contrast, burst speed was significantly affected by growth rate (*P* < 0.01, Wald’s test) but not affected by SL within the size range of the present samples (Table 1; see Table S3 for details). The Akaike information criterion (AIC) values of the linear, quadratic, and cubic polynomials fitted to the plots of growth rate against burst speed were 2000.5, 1996.9, and 1997.9, respectively (Table 2; see Table S4 for details). The quadratic function, which showed the smallest AIC value and had linear and quadratic coefficients significantly different from zero (Table 2; Wald’s test, *P* < 0.05), was selected as the best to describe the data (Fig. 2). The individuals of 20 dph (fast growers) showed clearly higher burst speeds than the same-size individuals of 30 dph (slow growers) (Fig. S1; *P* < 0.01).

**Table 1:** Summary of modeling results of generalized linear mixed models (GLMMs) and Wald’s test to examine effects of standard length (SL) and growth rate on routine or burst swimming speed (*n* = 171 and 161, respectively)

Relationship	Estimate	SE	*z* value	*P* value
Routine speed–SL	0.033	0.017	1.909	0.056
Routine speed–growth rate	− 0.166	0.297	− 0.560	0.575
Burst speed–SL	− 0.012	0.014	− 0.859	0.390
Burst speed–growth rate	0.729	0.238	3.063	0.002

The GLMMs were based on a Gamma distribution

**Table 2:** Summary of modeling results of generalized linear mixed models (GLMMs) and Wald’s test to examine the relationship between burst speed and growth rate (*n* = 161)

Model	Explanatory variable	Estimate	SE	*z* value	*P* value
Linear	Growth rate	0.640	0.193	3.310	< 0.001
Quadratic	Growth rate	4.042	1.411	2.864	0.004
	Growth rate^2^	–2 .229	0.918	− 2.428	0.015
Cubic	Growth rate	12.317	8.040	1.532	0.126
	Growth rate^2^	– 13.725	11.038	− 1.244	0.214
	Growth rate^3^	5.106	4.886	1.045	0.296

The GLMMs were based on a Gamma distribution

Akaike information criterion (AIC) values of the models; Linear: 2000.5, Quadratic: 1996.9, Cubic: 1997.9

### Relationships between morphological traits, growth rate, and burst speed

**Fig. 3:**
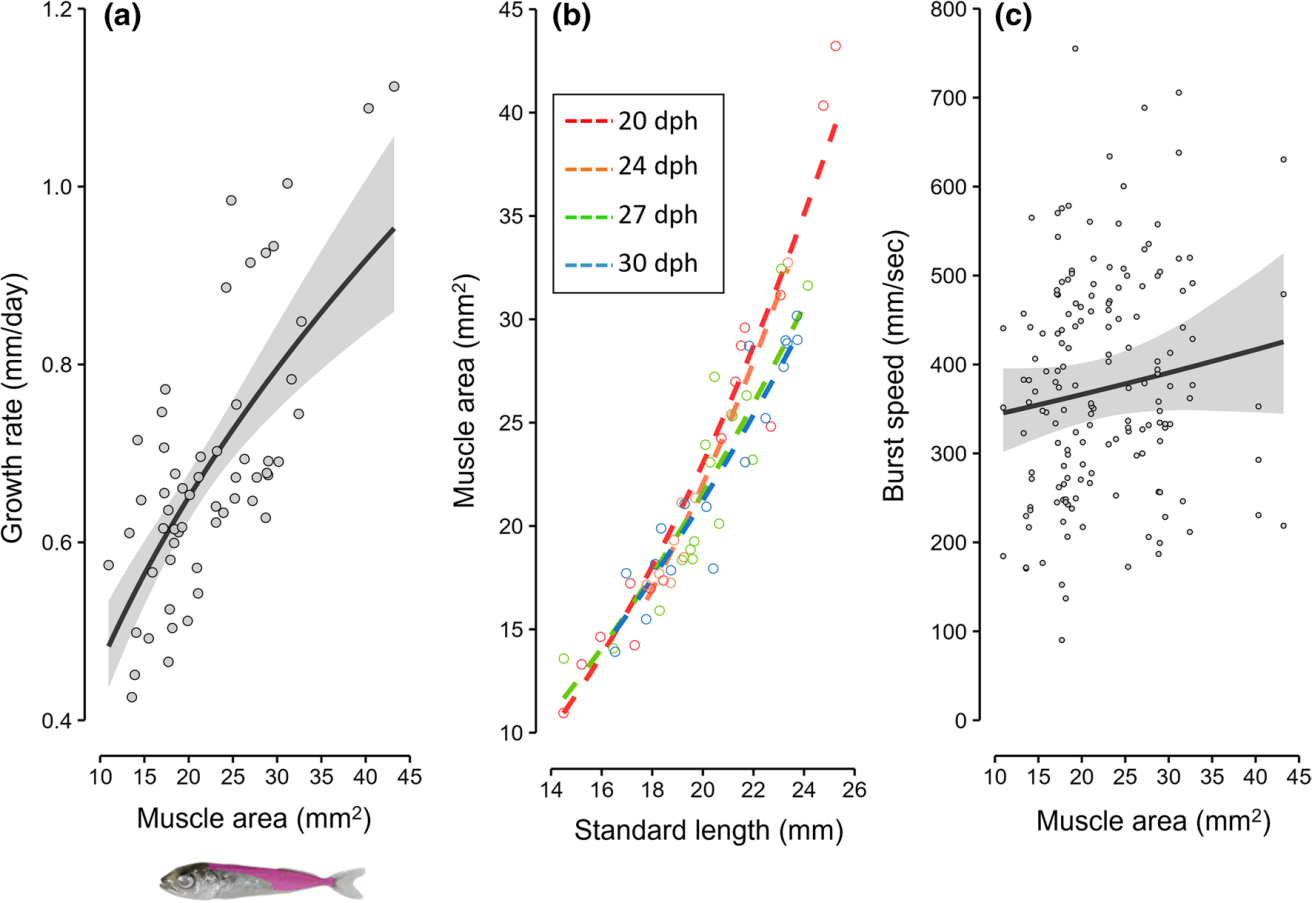
Relationship between growth rate and muscle area (**a**), muscle area and standard length or days post-hatch (dph) (**b**), and burst speed and muscle area (**c**). Solid and dashed lines indicate the generalized linear models (GLMs; **a** and **b**; *n* = 57) or the generalized linear mixed model (GLMM; c; *n* = 161) fitted to the data. Gray shadings on either side of the lines indicate the 95% confidence interval. All models were based on a Gamma distribution

Growth rate had a significant positive effect on muscle area (*P* < 0.01; Fig. 3a) but not on body depth (*P* = 0.53). Muscle area was significantly affected by both SL and dph (Fig. 3b). Larger individuals showed larger muscle areas (*P* < 0.01) and individuals of 20 dph showed larger muscle areas than the same-size individuals of 30 dph (*P* = 0.04). Individuals with larger muscle areas tended to show higher burst speeds even though the relationship was not significant (*P* = 0.16; Fig. 3c).

## Discussion

The most significant finding from these results is the positive correlation between growth rate and locomotor performance in the pelagic fish species, which has never been empirically demonstrated before. Fast growers of chub mackerel juveniles showed clearly higher burst speeds than the same-size individuals of slow growers. As the ration level was kept constant at a very high rate throughout the experiment, the differences in growth rate between individuals did not seem to be the result of differences in competitive ability which would mask any underlying relationship. The fact that burst speed, an indicator of escape speed, was positively related to a wide range of possible growth rates is consistent with our hypothesis as well as field observations that reported selective survival of fast-growing individuals of this species (Kamimura et al. 2015; Taga et al. 2019). Several studies have reported seemingly similar results. For example, Gregory and Wood (1999) reported a positive growth–swimming relationship in juvenile rainbow trout (*Oncorhynchus mykiss*) that was kept at a very low ration level. However, their analysis also detected a significant effect of body size on swimming performance. In addition, they also reported that smaller individuals of the experimental group had more fin damage than larger individuals. These effects of body size and fin damage make it unclear whether there is a significant positive effect of high growth itself, independent of body size or fin damage, on swimming performance in rainbow trout. Hence, the novelty of the present study lies in showing the positive growth–locomotor relationship by excluding the effect of body size or any other possible contributing factors.

The dome-shaped quadratic function best described the growth–burst relationship among the three tested models, suggesting that the relationship there between is non-linear. If the dome-shaped function truly represents the relationship, it implies that the erosion of energy resource for the development of locomotor performance by the investment for the acceleration of growth is evident as growth rate approaches 0.9 mm/day. Given that the total amount of energy acquired by an individual is limited, the dome-shaped relationship that defines the threshold of trade-off between investment for growth and investment for locomotor performance seems to be reasonable. However, due to the limited amount of data on extremely fast growers, the existence of trade-off between high growth and locomotor performance remains unclear. Further studies that compare the fitness of a dome-shaped function and that of a function with a horizontal asymptote using a dataset that contains substantial data on very fast growers would help clarify this point.

Our finding is distinct from the previous laboratory experiments that reported negative relationships only (Farrell et al. 1997; Billerbeck et al. 2001; Arendt 2003; Munch and Conover 2004; Lee et al. 2010, 2016) or non-relationships (Álvarez and Metcalfe 2007; Lindgren et al. 2018) between these two traits. The relationship we discovered can be regarded as a variation of the non-linear relationship between growth rate and swimming performance which was reported by Munch and Conover (2004). Both studies showed that a convex upward function best describes the relationship between the two traits. The function of Munch and Conover (2004), however, represents a clear negative relationship because its vertex is theoretically at zero growth, whereas the vertex of our function is at approximately 0.9 mm/day. In addition, the fact that the significant relationships between the two traits found in the above-mentioned previous studies were all negative suggests that at least another adaptive trait that positively relates with growth rate has to be assumed to explain the evolution of submaximal growth (otherwise, only minimal growth can evolve). The dome-shaped relationship implies that it is logically possible that submaximal growth evolves simply in response to trade-offs between growth rate and swimming performance.

Selection pressures that have led to the evolution of the substantially positive growth–swimming correlation in chub mackerel may be associated with their predation avoidance strategies in tight schools. As discussed by Hamilton (1971), schooling is particularly evident in fish species that inhabit open waters, including juvenile chub mackerel. Because stragglers are known to suffer a higher risk of predation than school members (Parrish 1989), and an individual’s location in the school is likely to have a significant effect on survival from predatory attack (Hamilton 1971; Parrish 1989), individuals of gregarious prey species are predicted to struggle to avoid isolation from and win the optimal location in the school that consists of selfishly behaving conspecifics. In addition, given that severe cannibalism is often observed in captivity (Meguro 2002), chub mackerel larvae and juveniles seemingly have to exert additional effort to avoid being eaten by other members of the school, for which higher swimming speed should be especially important. Swimming performance is a key trait that affects the odds of success in these attempts. Under such conditions, natural selection can favor individuals whose growth rate trades off with some trait other than swimming performance unless it is extremely high, which may lead to the evolution of unique energy allocation strategies in chub mackerel. Atlantic silversides are also known to use offshore areas for overwintering at the adult stage; nevertheless, several previous studies reported simple trade-offs between growth rate and swimming performance. However, they spend most of their lives in estuarine and coastal areas; even during winter, most of the population stay in shallow regions no deeper than 50 m (Fay et al. 1983). Therefore, they would still be able to use fixed shelters on the seafloor to avoid predation at the overwintering site. Thus, hiding into some kind of immobile shelter is likely to be a lifelong effective predator-avoidance strategy for Atlantic silversides. This is not the case for chub mackerel; their main habitat is so deep that it is impossible to utilize the seafloor for evacuation, and the almost only shelter available to them is the other members of school. Whether a species can use immobile shelters as at least one of the primary measures to avoid predation may be an important factor that determines whether a simple trade-off between growth rate and locomotor ability can evolve in the species.

At present, however, it is difficult to discern whether our results showed a rare species- or habitat-specific relationship or a fundamental relationship common to diverse animals, including at least some of the previously tested species. This is because the related studies did not use a unified experimental design that would allow a stringent comparison with our data. Most of the previous studies that identified trade-offs between growth rate and locomotor performance were based on group comparison between fast- and slow-growing phenotypes (e.g. Kolok and Oris 1995; Gregory and Wood 1998, 1999; Billerbeck et al. 2001; Lee et al. 2010), and only the present study on chub mackerel and Munch and Conover (2004) on Atlantic silverside determined the relationship between these two traits as a single continuous function at an individual level. In addition, the index of swimming performance that Munch and Conover (2004) used was critical swimming speed, indicative of aerobic performance representing stamina rather than escape speed. Moreover, some of the previous studies standardized swimming speeds in terms of body lengths per second, whereas we used un-scaled swimming speeds, which seemed to represent the escape ability of the prey animal most directly. Therefore, we are left with the possibility that dome-shaped curves might apply to the previously tested species, even to the most well-studied Atlantic silverside, if their growth–swimming relationship was determined as a continuous function of growth rate and un-scaled actual burst speed. In any case, experimental verification using not only laboratory animals that inhabit closed water or coastal areas but also non-laboratory animals that inhabit various types of habitats will be required for a proper understanding of the principles underlying the evolution of growth–swimming relationship.

Growth rate did not affect routine speed but affected burst speed. This result would be explained by the difference between the roles of these two types of swimming performance. Routine speed would represent the swimming performance of chub mackerel under their normal conditions, whereas burst speed would represent the maximum potential performance under some urgent conditions, such as when they are attacked by predators or about to capture prey. Thus, burst speed is likely to require higher and more instantaneous energy than routine speed, which may be a possible reason why growth rate was positively related to burst speed, not routine speed. In any case, growth–swimming relationships could differ dramatically depending on the type of swimming performance.

Muscle area also showed a clearly positive correlation with growth rate, and rapidly growing individuals showed larger muscle areas than the same-size slow growers (Fig. 3a, b). As it is reasonable to assume that burst performance is underpinned by muscle to some extent, the fact that fast growers had larger muscle areas may partly explain the positive effect of fast growth on burst speed. However, the relationship between muscle area and burst speed was not significant. In addition, the possible trade-offs between growth rate and burst speed observed in very fast growers were not explained by the relationship between growth and muscle area. Therefore, further physiological and/or extended morphological studies will be needed to understand the mechanisms underlying the non-linear relationship between growth rate and locomotor performance in this species. As another important next step, biological factors that yield and trade-off with these morphological advantages of growth (i.e. larger body size and larger muscle area at a given age) have to be clarified. We hypothesize that these advantages of growth may be accomplished by higher food intake and/or higher food conversion efficiency (Present and Conover 1992) and may trade-off with higher risk of predation during aggressive foraging by overwhelming predators that cancel the locomotor advantage (Takasuka et al. 2007) and/or tolerance of feed deprivation (Dupont-Prinet et al. 2010). Identifying these factors is difficult, but essential if we are to understand the evolutionary mechanism of this newly found growth strategy.

In conclusion, the present study is the first to show a positive effect of growth rate itself on swimming performance in aquatic vertebrates under experimental conditions. The substantially positive growth–swimming relationship in chub mackerel is consistent with field observations showing selective survival of fast-growing individuals of this species, reconciling the current contradiction between laboratory experiments on coastal- or freshwater-living animal species and field observations on pelagic fishes. This finding strongly suggests the importance of experimental verification using animals that inhabit various types of habitats in understanding the principles underlying the evolution of growth–locomotor relationship. Our detailed methodology also highlights the potential risk associated with experiments that are based only on group comparison: resultant relationships, which can be monotone-increasing, monotone-decreasing, or non-significant depending on the observed range of growth rate, may lead to a simplistic interpretation of growth–swimming relationship. Further experimental studies on various species which examine the relationship between growth rate and any trait that correlates therewith as a continuous function should provide a better understanding of the mechanisms underlying the evolution of a broad range of growth strategies.

## Supplementary Information

Below is the link to the electronic supplementary material.

Supplementary file1 (DOCX 106 kb)

## Data Availability

Basic data of individuals used in analysis are deposited in Table S1 in the supplemental materials. Raw data will also be made available in the DRYAD Digital Depository (https://doi.org/10.5061/dryad.bzkh1896k).

## Code availability

R codes used for analysis is also available in the DRYAD Digital Depository (https://doi.org/10.5061/dryad.bzkh1896k).
