# Supplementary material for: Positive effects of fast growth on locomotor performance in pelagic fish juveniles
Source: Oecologia. 2022 Jul 4;199(3):589–97. doi: 10.1007/s00442-022-05216-6 (PMC9309151; doi:10.1007/s00442-022-05216-6)
Supplement: Supplementary file 1 — Supplementary file1 (DOCX 106 kb) [file 442_2022_5216_MOESM1_ESM.docx]

**Electronic supplemental material**

**Positive effects of fast growth on locomotor performance in pelagic fish juveniles**

Masahiro Nakamura^1*^, Michio Yoneda^1^, Taizo Morioka^1^, Akinori Takasuka^2^, Nozomi Nishiumi^3^

*^1^Fisheries Technology Institute, Japan Fisheries Research and Education Agency*

*Hakatajima Filed Station, Imabari, Ehime 794-2305, Japan*

*^2^Graduate School of Agricultural and Life Sciences, the University of Tokyo, Bunkyo, Tokyo 113-8657, Japan*

*^3^National Institute for Basic Biology, Higashiyama 5-1, Myodaiji, Okazaki, Aichi 444-8787, Japan*

*Corresponding author: M. Nakamura

Tel: +81-897-72-0204, Fax: +81-897-72-2544

Email: mnakamura@affrc.go.jp

**Table S1:** Standard length (SL), days post hatch (dph) and rearing tanks for individuals used for analysis

| ID | SL (mm) | dph | tank |  | ID | SL (mm) | dph | tank |
| --- | --- | --- | --- | --- | --- | --- | --- | --- |
| 1 | 21.51 | 20 | A |  | 40 | 16.46 | 27 | D |
| 2 | 22.69 | 20 | A |  | 41 | 17.76 | 30 | A |
| 3 | 17.13 | 20 | A |  | 42 | 16.97 | 30 | A |
| 4 | 25.25 | 20 | B |  | 43 | 23.19 | 30 | A |
| 5 | 23.07 | 20 | B |  | 44 | 20.14 | 30 | A |
| 6 | 18.44 | 20 | B |  | 45 | 22.48 | 30 | A |
| 7 | 15.95 | 20 | B |  | 46 | 23.27 | 30 | B |
| 8 | 24.76 | 20 | C |  | 47 | 23.34 | 30 | B |
| 9 | 21.66 | 20 | C |  | 48 | 21.83 | 30 | B |
| 10 | 14.49 | 20 | C |  | 49 | 23.72 | 30 | B |
| 11 | 15.21 | 20 | C |  | 50 | 21.67 | 30 | B |
| 12 | 21.29 | 20 | D |  | 51 | 18.74 | 30 | C |
| 13 | 20.73 | 20 | D |  | 52 | 16.53 | 30 | C |
| 14 | 17.93 | 20 | D |  | 53 | 23.74 | 30 | C |
| 15 | 17.30 | 20 | D |  | 54 | 18.35 | 30 | C |
| 16 | 23.36 | 24 | A |  | 55 | 19.28 | 30 | D |
| 17 | 19.25 | 24 | A |  | 56 | 20.41 | 30 | D |
| 18 | 19.71 | 24 | A |  | 57 | 18.11 | 30 | D |
| 19 | 21.12 | 24 | A |  |  |  |  |  |
| 20 | 18.27 | 24 | B |  |  |  |  |  |
| 21 | 18.73 | 24 | B |  |  |  |  |  |
| 22 | 18.86 | 24 | B |  |  |  |  |  |
| 23 | 17.78 | 24 | B |  |  |  |  |  |
| 24 | 19.16 | 24 | B |  |  |  |  |  |
| 25 | 21.73 | 27 | A |  |  |  |  |  |
| 26 | 20.46 | 27 | A |  |  |  |  |  |
| 27 | 21.97 | 27 | A |  |  |  |  |  |
| 28 | 19.51 | 27 | A |  |  |  |  |  |
| 29 | 20.64 | 27 | B |  |  |  |  |  |
| 30 | 24.15 | 27 | B |  |  |  |  |  |
| 31 | 23.10 | 27 | B |  |  |  |  |  |
| 32 | 21.17 | 27 | B |  |  |  |  |  |
| 33 | 19.60 | 27 | C |  |  |  |  |  |
| 34 | 20.10 | 27 | C |  |  |  |  |  |
| 35 | 14.50 | 27 | C |  |  |  |  |  |
| 36 | 20.29 | 27 | C |  |  |  |  |  |
| 37 | 18.29 | 27 | D |  |  |  |  |  |
| 38 | 19.66 | 27 | D |  |  |  |  |  |
| 39 | 19.18 | 27 | D |  |  |  |  |  |

**Fig. S1:** Relationships between burst speed and standard length (SL) at each day post hatch (dph). Generalized linear model (GLM) was used to determine whether burst speed was significantly affected by dph, which contained mean burst speed of individuals as a response variable and SL and dph as explanatory variables. Dashed lines indicate generalized linear models fitted to the data at each dph-group. Individuals of 30 dph (slow growers) were estimated to show significantly low burst speed compared to the same-size individuals of 20 dph (*P* < 0.01). Significant effect of SL on burst speed was detected in this analysis (*P* < 0.01). The GLM was based on a Gamma distribution


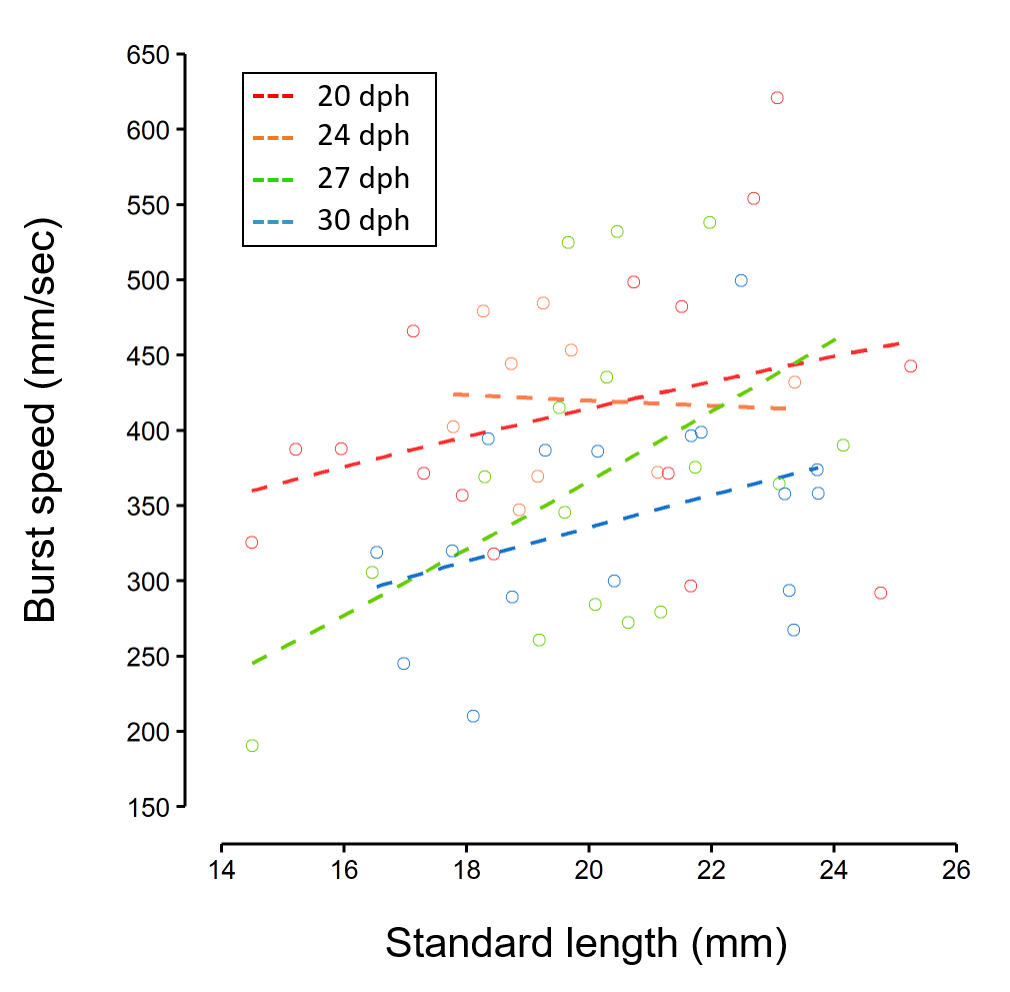


**Table S2: Summary of results of a generalized linear mixed model (GLMM) and Wald’s test used to examine whether routine speed was significantly affected by standard length (SL), growth rate, and aliz arin complexone (ALC) marking.** The GLMM was based on a Gamma distribution. It contained SL, growth rate, and ALC marking as explanatory variables and individual ID and rearing tank as random effects. VIF = variance inflation factor, SE = standard error

| Explanatory variable | VIF | Estimate | SE | z value | *P* value |
| --- | --- | --- | --- | --- | --- |
| SL | 1.75 | 0.033 | 0.017 | 1.909 | 0.056 |
| growth rate | 1.74 | -0.166 | 0.297 | -0.560 | 0.575 |
| ALC marking | 1.01 | -0.039 | 0.067 | -0.573 | 0.567 |
|  |  |  |  |  |  |
| Random effects | variance | SD |  |  |  |
| individual id | 0.05 | 0.23 |  |  |  |
| rearing tank | < 0.01 | < 0.01 |  |  |  |

**Table S3: Summary of results of a** **generalized linear mixed model (GLMM) and Wald’s test used to examine whether burst speed was significantly affected by standard length (SL), growth rate, and alizarin complexone (ALC) marking.** The GLMM was based on a Gamma distribution. It contained SL, growth rate, and ALC marking as explanatory variables and individual ID and rearing tank as random effects. VIF = variance inflation factor, SE = standard error

| Explanatory variables | VIF | Estimate | SE | z value | *P* value |
| --- | --- | --- | --- | --- | --- |
| SL | 1.70 | -0.012 | 0.014 | -0.859 | 0.390 |
| growth rate | 1.66 | 0.729 | 0.238 | 3.063 | 0.002 |
| ALC marking | 1.03 | 0.109 | 0.060 | 1.817 | 0.069 |
|  |  |  |  |  |  |
| Random effects | variance | SD |  |  |  |
| indvidual id | 0.01 | 0.08 |  |  |  |
| rearing tank | 0.01 | 0.08 |  |  |  |

**Table S4: Details of modeling results of generalized linear mixed models (GLMMs) and Wald’s test to examine the relationship between burst speed and growth rate.** The GLMMs were based on a Gamma distribution, and contained growth rate as an explanatory variable and individual ID and rearing tank as random effects. SE: standard error; SD: standard deviation

| Model | Explanatory variable | Estimate | SE | z value | P value |
| --- | --- | --- | --- | --- | --- |
| Linear | Growth rate | 0.6395 | 0.1932 | 3.31 | 0.00093 |
| Quadratic | Growth rate | 4.0419 | 1.4114 | 2.864 | 0.00419 |
|  | Growth rate^2^ | -2.2288 | 0.9178 | -2.428 | 0.01517 |
| Cubic | Growth rate | 12.317 | 8.04 | 1.532 | 0.126 |
|  | Growth rate^2^ | -13.725 | 11.038 | -1.244 | 0.214 |
|  | Growth rate^3^ | 5.106 | 4.886 | 1.045 | 0.296 |
|  |  |  |  |  |  |
| Model | Random effects | Variance | SD |  |  |
| Linear | individual ID | 0.010312 | 0.10155 |  |  |
|  | rearing tank | 0.003845 | 0.06201 |  |  |
| Quadratic | individual ID | 0.00772 | 0.08786 |  |  |
|  | rearing tank | 0.001724 | 0.04152 |  |  |
| Cubic | individual ID | 0.006845 | 0.08274 |  |  |
|  | rearing tank | 0.002291 | 0.04786 |  |  |
| Akaike information criterion (AIC) values of the models; Linear: 2000.5, Quadratic: 1996.9, Cubic: 1997.9 | | | | | |
